# Prevalence of Feline Immunodeficiency Virus and *Toxoplasma gondii* in Feral Cats on St. Kitts, West Indies

**DOI:** 10.3390/vetsci8020016

**Published:** 2021-01-21

**Authors:** Xinyu Chi, Kexin Fang, Liza Koster, Jevan Christie, Chaoqun Yao

**Affiliations:** 1 Departments of Biomedical Sciences, Ross University School of Veterinary Medicine, P.O. Box 334, Basseterre 00265, Saint Kitts and Nevis; xinyuc.20@intl.zju.edu.cn (X.C.); kxfang@zju.edu.cn (K.F.); 2 Departments of One Health Center for Zoonoses and Tropical Veterinary Medicine, Ross University School of Veterinary Medicine, P.O. Box 334, Basseterre 00265, Saint Kitts and Nevis; lkoster@utk.edu (L.K.); jevanchristie@gmail.com (J.C.); 3 Departments of Clinical Sciences, Ross University School of Veterinary Medicine, P.O. Box 334, Basseterre 00265, Saint Kitts and Nevis

**Keywords:** *Toxoplasma gondii*, oocyst shedding, domestic cat, feral cat, feline immunodeficiency virus, feline leukemia virus

## Abstract

*Toxoplasma gondii* (*T. gondii*) is a cosmopolitan protozoan parasite that infects all warm-blooded species including humans. The definitive hosts of *T. gondii* are felid vertebrates including the domestic cat. Domestic cats shed oocysts for approximately two weeks in their feces after the primary infection. It has been shown that feline immunodeficiency virus (FIV) positive cats have a higher prevalence of and a higher titer of antibodies to *T. gondii* than those of FIV-negative cats. The main purposes of this study were to determine FIV prevalence and to investigate the oocysts shedding in FIV-positive and FIV-negative feral cats on St. Kitts. Fecal samples were collected from feral cats while their FIV statues were determined using a commercial SNAP kit. Total fecal DNA of each cat was tested for the presence of *T. gondii* DNA using a polymerase chain reaction (PCR) consistently detecting one genome equivalent. A FIV-positive status was detected in 18 of 105 (17.1%, 95% confidence interval (CI): 9.9%−24.3%) feral cats sampled. Furthermore, males were three times more likely to be FIV positive than females (*p* = 0.017) with an odds ratio of 3.93 (95% CI: 1.20–12.89). Adults were found to have at least twice the prevalence of FIV compared to cats younger than one year of age (*p* = 0.056) with an odds ratio of 3.07 (95% CI: 0.94–10.00). *Toxoplasma gondii* DNA was not detected in the feces of any of the 18 FIV-positive (95% CI: 0%−0.18%) and 87 FIV-negative cats (95% CI: 0%−0.04%). A follow-up study with a much bigger sample size is needed to prove or disprove the hypothesis that FIV-positive cats have a higher prevalence of shedding *T. gondii* oocysts than FIV-negative cats.

## 1. Introduction

*Toxoplasma gondii*, a cosmopolitan protozoan, requires an intermediate host and a definitive host in its life cycle. The intermediate host covers all warm-blooded vertebrates including humans whereas the definitive host is narrowly limited to the domestic cat (*Felis catus*) and other felids. A definitive host may shed oocysts in its feces for approximately two weeks upon the primary infection. Specifically it releases the oocysts in its feces for 3–10 days, ≥18 days and ≥13 days respectively, for being infected by ingestion of tissue cysts with bradyzoites, oocysts or tachyzoites [1]. Upon infection by tissue cysts with bradyzoites, a domestic cat starts oocyst shedding as early as four days post infection (PI) and ends at Day 16 PI with a major peak between the day 5 and day 7 PI [2,3,4].

Oocyst shedding by cats and the resultant environmental contamination with oocysts play an important role in the high prevalence of *T. gondii* among various intermediate hosts worldwide because one of the major routes of infection for the intermediate host is by oral ingestion of infective oocysts [5,6]. It is widely accepted that a definitive host sheds oocysts for approximately two weeks when it is initially infected thereafter it no longer sheds oocysts upon secondary (subsequent) infection due to a long-lasting immunity to prevent oocyst shedding. Limited laboratory data have also suggested that oocyst shedding occurs after secondary infection with the same or different strains of the primary infection although with a much lower number of oocysts shed [4,7]. Furthermore, it has been shown in the laboratory that cats with primary *T. gondii* infection that have stopped oocyst shedding start to re-shed oocysts again starting 20–21 days after immunosuppression that is induced by daily parenteral administration of dexamethasone (1 mg/kg/day) for 30 days. This re-shedding lasts 9–10 days [8]. However, whether a definitive host with immunity as a result of a prior exposure starts to re-shed oocysts once its immunity is compromised needs to be tested and confirmed in feline populations. Feline immunodeficiency virus (FIV) eventually causes an inability of its host’s immune system to mount an effective cell-mediated immune response, resulting in marked immune dysfunction with severe and progressive respiratory and intestinal disease in its host [9]. It has been showed that FIV-positive urban stray cats have a higher prevalence and higher titer of antibodies to *T. gondii* compared to FIV-negative cats [10,11]. It has been found that 84.9% of domestic cats on St. Kitts have antibodies to *T. gondii* [12] and 16.5% of cats on St Kitts are FIV positive [13]. It is likely that FIV infection in a domestic cat changes the dynamics of its oocyst shedding with *T. gondii* infection rendering it to re-shed oocysts and/or shed oocysts for an extended period of time, leading to an increase in *T. gondii* prevalence in feces among FIV-positive cats in comparison with FIV-negative ones. The main objective of the current study was to investigate fecal oocyst shedding in the FIV-positive versus FIV-negative feral cats. An additional aim was to give an update of FIV prevalence on St. Kitts as previous studies had existed.

## 2. Materials and Methods

### 2.1. Ethical Approval

Animal use of the study was approved by the Institutional Animal Care and Use Committee (IACUC) of Ross University School of Veterinary Medicine with the approval IACUC number (15-1-001).

### 2.2. Sample Size

The samples size was calculated using the following formula: sample size n = [Z_1−α/2_^2^ × p × (1 − p)]/d^2^ as previously described [14]. For the current study, we assumed the prevalence of 2.5% and 5% of *T. gondii* DNA in the feces of FIV-negative and FIV-positive cats, respectively. We wanted to calculate sample size with the precision/absolute error of 10% (d = 0.1) and at type I error of 5% (Z_1−α/2_ = 1.96). Therefore, for FIV-negative cats: n = (1.96^2^ × 0.025 × 0.975)/0.1^2^ = 9 samples; for FIV-positive cats: n = (1.96^2^ × 0.05 × 0.95)/0.1^2^ = 18 samples.

### 2.3. Data and Sample Collection

Feral cats were caught between September 2014 and July 2015 on St. Kitts. They were all included in a trap–neuter–return program entitled the ‘Feral Cat Project’ (FCP). The FCP was started years ago and has been continuously carried out by the Ross University Veterinary Clinic (RUVC) in order to manage and control the population of the feral cats on the island. Feral cats were caught at 15 locations at Basseterre and adjacent areas including new road, Texaco Gas station, Matingly, Lime Kiln, Half Moon Bay, Calypso Bay, Bird Rock, Depay Bay, food center, Camps, Horizons Villa, Basseterre and West Farms. They were brought to RUVC where their sex and age were determined prior to neutering. Their ages were determined as young if less than one year of age or adult if more than one year of age based on dentition, body weight and stature. Upon anesthesia blood and feces were collected from each cat prior to their neutering and the FIV-positive cats were euthanized. Euthanasia was performed while the cat was under general anesthesia by injecting a barbituric acid derivative intravenously that is, pentobarbital as a single agent at 120 mg/kg. Death was confirmed by a veterinarian by absence of a corneal reflex, apnea and heartbeat. Euthanasia was strictly limited to a period of two years to study the long-term effect of removal of FIV-positive cats on general FIV prevalence on St. Kitts, which constitutes a MSc thesis. All cats in the FCP were tested for FIV antibodies using a commercially available patient-side test (SNAP FIV/FeLV Combo Test, IDEXX Laboratories, Westbrook, ME, USA) as per the manufacturer’s instruction. Colon flush was collected after each cat was infused 3 mL veterinary saline (Abbott Laboratory, Abbott Park, IL, USA) using a lubricated Kendall Feeding Tube and Urethral Catheter (Covidien, LLC., Mansfield, MA, USA) connected to a 5 mL syringe. Approximately one gram of feces was taken from the anus using a gloved finger in the case of very firm stool. The fecal samples were frozen immediately and kept at −80 °C prior to DNA extraction.

### 2.4. Detection of Toxoplasma gondii

Total DNA was individually extracted from each feces using QIAamp DNA Stool Kit (QIAGEN, Germantown, MD, USA) according to the manufacturer’s instructions. DNA quality and quantity were monitored with the Tecan Infinite M200 Pro (Tecan, Mannedorf, Switzerland) or NanoPhotometer (Implen, Westlake Village, CA, USA). All DNAs were kept at −20 °C until use. To detect the DNA of *T. gondii* polymerase chain reaction (PCR) was performed to target the 529-base pair (bp) repeat element of *T. gondii* using the primer pair Tox-9F (5′AGGAGAGATATCGGGACTGTAG3′) and Tox-11R (5′GCGTCGTCTCGTCTAGATCG3′) with an expected amplicon of 163 bp. The method was chosen due to its high sensitivity, that is, it has been reported quantitative PCR (qPCR) amplification of the element consistently detects one genome equivalent (80 fg DNA) per sample and is still positive in 7 out of 30 samples when the level of DNA is as low as 0.8 fg DNA [15,16]. All PCR was performed using HotStart Taq Plus 2×Master Mix (QIAGEN) with 0.5 μM final concentration of each forward and reverse primer as previously described [17]. Briefly, 9.8 μL of each fecal DNA extracts was used as a template in 20 μL PCR mix. PCR was carried out in a thermal cycler (Mastercycler Nexus Gradient, Eppendorf, Hauppauge, NY, USA) with the following parameters: initial denaturing at 95 °C 2 min, 45 cycles of 95 °C 30 s, 58 °C 30 s and 72 °C 1 min followed by a final extension at 72 °C for 10 min. DNA of the RH strain (genotype I) of *T. gondii* and molecular grade water were used as positive and negative controls, respectively. The amount of positive control DNA used in each PCR was 27.4 fg. The PCR amplicons were detected by electrophoresis of the all mix on a 1.5% agarose gel using a 100-bp ladder (Invitrogen, Carlsbad, CA, USA) as a size marker. Positive samples were repeatedly tested and were deemed false positive provided that the second PCR was negative.

## 3. Results

### 3.1. FIV Prevalence of Feral Cats on St. Kitts

Between September 2014 and July 2015 a total of 105 feral cats were sampled. The overall prevalence of FIV was 17.1% (18/105). More specifically FIV prevalence was 25.5% (14/55) for males and 8.0% (4/50) for females. The odds ratio (OR) for being male was 3.93 (95% confidence interval (CI): 1.20–12.89). FIV prevalence was 11.5% for the cats younger than 1 year old (6/53) and 28.8% for the cats were over 1 year old (8/28). OR for being the latter was 3.07 (95% CI: 0.94–10.00) (Table 1).

All 105 feral cats were tested negative for feline leukemia virus (FeLV) antigens, which is consistent with earlier reports that St. Kitts cats are FeLV free [18,19] and will not be further discussed.

### 3.2. Failure to Detect T. gondii DNA in Feral Cats’ Feces

Among 18 FIV-positive and 87 FIV-negative feral cats, none of their feces were found to be *T. gondii* positive by PCR. The 95% CI of *T. gondii* prevalence for the FIV-positive and -negative cats were 0−0.18% and 0−0.04%, respectively. The 95% CI for the overall prevalence of *T. gondii* of the feral cat population included in the current study ignoring their FIV status was 0.00−0.03%.

## 4. Discussion

The purpose of the current study was to detect oocyst shedding among feral cats on St. Kitts by taking advantage of the high FIV prevalence among them [13]. Neither the 18 FIV-positive nor the 87 FIV-negative cats on St. Kitts that were tested for *T. gondii* using the highly sensitive PCR amplification of the 529-bp repeat element were found to be positive for shedding this protozoan. It has been reported that this molecular technique is capable of constantly detecting one genome equivalent, that is, 80 fg DNA and approximately one-quarter times with one hundredth genome equivalent of *T. gondii* [15,16]. We used one-third genome equivalent DNA as positive controls and it worked consistently. Therefore, it is of high confidence to state that the estimate prevalence of oocyst shedding of *T. gondii* in feral cats on St. Kitts, no matter whether FIV-positive or FIV-negative is close to zero (95% CI: 0.00−0.03%). It is worth pointing out that not all the stools during a cat’s window of oocyst shedding would have contained *T. gondii* oocysts because the oocyst release is not continuous. Furthermore, an immunocompetent cat sheds oocysts for approximately only two weeks during the primary infection in its entire life [1,2,3,4]. This combination of a narrow oocyst shedding window and discontinuous oocyst shedding greatly reduce the probability to get a positive fecal sample in an infected cat.

In general, oocyst shedding is low among domestic cats. In a survey of feral cat feces by fecal flotation with a detection limit of 250 oocysts/g of feces, one of 78 samples collected on Prince Edward Island, Canada was found to contain *T. gondii* oocysts [20]. In Sao Paula State, Brazil, the prevalence of oocysts among client owned domestic cats was 1.3% (3/237) determined by microscopy and bioassay [21]. In the Morro Bay area of California, it was 0.9% (3/326). Specifically, it was 1.3% (2/153) in client owned cats and 0.9% (1/107) in feral cats [22]. Among 61,224 fecal samples of client owned pet cats submitted to IDEXX for diagnosis 84 (0.14%) were found to be positive with *T. gondii* DNA by PCR. In a similar survey, 0.11% (26/24,106) of fecal samples of client owned domestic cats from Germany, Austria, France and Switzerland were positive [23,24]. It should also be cautious that not all positive results of PCR for detecting *T. gondii* DNA in cat feces is from oocysts. Those cats who ingested a *T. gondii*-infected prey were always found to be PCR positive one day after ingestion, this positive finding may even extend to the following two days [25].

Between September 2014 and July 2015, an overall FIV prevalence of 17.1% was found among the feral cats at 15 different locations near Basseterre, St. Kitts. Earlier reports of FIV prevalence on the same island showed 15.2% in 2006–2007, 13.9% in 2009 and 36.4% in 2010 in feral cat populations [18,19]. The much higher prevalence in 2010 might be due to small sample size of only 11 cats [19]. The FIV prevalence among feral cats on St. Kitts between 2006 and 2015 apparently maintained a constant level of approximately 15% despite of continuous effort of FCP to minimize mating and aggression by neutering. The current reported rate was similar to that of feral cat population in Germany [26]. It is much higher than that of feral cat populations in many regions such as Ethiopia [27], Portugal [28,29], Korea [30], Florida [31,32] Ottawa, Canada [33], Prince Edward Island, Canada [20,34], Texas [35], Hawaii [36], UK [37], Belgium [11] and is lower than that of feral cat populations in UK [38], Egypt [39], Australia [40] and Grenada, West Indies [41] (Table 2). Furthermore, the prevalence was much higher in male cats compared to female cats with an OR 3.93 (95% CI: 1.20–12.89, Table 1). This is similar to what has been previously observed in the domestic cat. In South Australia, de-sexed males had a much lower prevalence than intact males. Both intact and de-sexed male had a higher prevalence than female cats. More specifically the prevalence in the de-sexed and intact males between the age of 5 and 9 years old was 17% and 27%, respectively while the prevalence difference in the groups between the age of 9 and 13 years old was 16% and 35%, respectively. In contrast, the prevalence for the same age group of de-sexed and intact female was 7% and 5%, 10% and 0%, respectively [42]. What causes male cats to have a higher prevalence than female cats remains to be determined in the feral population. It is hypothesized that the major mode of FIV transmission among the domestic cat is by the aggressive behavior such as biting between male cats [42]. However, it is also worth noting that no difference was detected between sexes for FIV prevalence in feral cats in the Barley Park Farm, UK [38]. In addition, the FIV prevalence of the feral cats one year old or older was at least double that of ones less than one year old with an OR 3.06 (95% CI: 0.94–10.00, Table 1). This may be due to behavioral and/or biological difference between kittens and adults.

One drawback of the study was a limited sample size with a possible type II error. Although sample sizes of both FIV-negative and FIV-positive feral cats met minimal estimates no cats were detected positive for *T. gondii*. This is surprising with the high seroprevalence of *T. gondii* in the feral cat population and in other domestic animals on St. Kitts. Three quarters of the feral cats carried antibodies to this parasite during two cross-sectional survey studies carried out in 2005–2006 by a modified agglutination test. In addition, 16–23% of pigs, sheep and goats were PCR positive for the parasite’s DNA in their heart tissues [12,41,44]. Another limitation of this study was the lack of titers of antibodies to FIV. Not all FIV-positive cats are immunocompromised. FIV-positive animals might take years to develop disease. It is plausible that the lack of oocytes shedding in FIV-positive cats could be because they have not reached an immunosuppressed status yet. A bigger samples size with their antibody titers determined would have certainly increased our chances of detecting *T. gondii* DNA in feral cats. The third limitation was omitting fecal flotation to detect *T. gondii* oocysts, which was due to not enough fecal material collected from all cats for such an analysis. Nevertheless, PCR employed here consistently detected *T. gondii* DNA when only approximately one-third of a genome DNA equivalent was used in positive controls. Lastly, the study subjects were not tested for antibodies to *T. gondii*, so their infection status were unknown. Therefore, it is impossible to discern whether a negative PCR for *T. gondii* DNA in feces is due to lack of oocyst shedding in a *T. gondii*-infected cat or lack of infection in an uninfected animal.

Finally, it is worthy of concisely discussing mortality in FIV infected cats with an active or reactivated *T. gondii* infection. Two fatal cases of myelitis in cats with FIV and *T. gondii* co-infection have been reported. The first case was a 10-year-old cat with progressive paralysis and a postmortem diagnosis was made with ultrastructural analysis and immunohistochemistry [45]. The second case was a 10-year-old cat suffering from reactivated toxoplasmosis, resulting in lameness of the right thoracic limb and bilateral pelvic limb paresis. Nevertheless, the cat had been vaccinated with FIV vaccine within the past 12 months [46]. Furthermore, it had been found under laboratory conditions that clinically normal cats carrying active FIV infections suffered from catastrophic general toxoplasmosis upon paratenic infection with *T. gondii* tachyzoites [47]. Consequently, it is plausible that FIV and *T. gondii* co-infected cats are removed from the feral cat population, which diminishes our capacity to detect the parasite.

## 5. Conclusions

The sampled feral cats on St. Kitts, irrespective of their FIV status were not detected shedding *T. gondii* oocysts as determined by a highly sensitive PCR, which was apparently hindered by a combination of narrow window of and discontinuous oocyst shedding of infected cats. In St. Kitts, male feral cats were three times more likely to be FIV positive than females and adults were two times more likely to be FIV positive compared to young cats. The zoonotic risk of exposure to *T. gondii* through feral cats, irrespective of their FIV status could not be accurately determined in the current study. A further study with a much larger sample size is warranted to test the hypothesis that FIV-positive cats have a higher prevalence than FIV-negative ones in shedding *T. gondii* oocysts.

## Figures and Tables

**Table 1:** Age, sex and the status of feline immunodeficiency virus (FIV) of feral cats tested in the study.

Age ^#^	FIV+	FIV–	Total	Prevalence (%)	95% CI	Odds Ratio	95% CI
<1 year	6	46	52	11.5	2.8–20.2		
≥1 year	8	20	28	28.8	12.0–45.6	3.06	0.94–10.00
ND	4	21	25	16.0	1.6–30.4		
Total	18	87	105	17.1	9.9–24.3		
**Sex** *							
Male	14	41	55	25.5	14.7–37.0	3.93	1.20–12.89
Female	4	46	50	8.0	0.5–15.5		
Total	18	87	105	17.1	9.9–24.3		

ND: Not determined. CI: confidence interval. #: χ^2^ statistic is 3.6573 between < 1 and ≥ 1 year group; *p* = 0.056. *: χ^2^ statistic is 5.6176; *p* = 0.018.

**Table 2:** The prevalence of feline immunodeficiency virus (FIV) among feral cats in different geographic regions.

Region	Cats Examined	FIV + Cats	Prevalence (%)	Year	References
**Europe**					
Ghent, Belgium	346	39	11.3	1998–2000	[11]
Berlin, Germany	39	5	15.4	1996–1999	[26]
Portugal	50 226	0 23	0.0 10.2	1992–2007 2003–2005	[29] [28]
Birmingham, UK Barley Park Farm, UK	517 55	54 26	10.4 47.3	1997	[37] [38]
**Africa**					
Addis Ababa, Ethiopia	41	0	0.0		[27]
Cairo, Egypt	174	59	33.9	2008–2009	[39]
**Asia**					
Seoul, Korea	66	2	3.0	2013	[30]
**North America**					
Key Largo, Florida			3.3	1999–2013	[31]
Northern Florida	553	29	5.2	1999–2000	[32]
College Station, Texas	155	10	6.5	1998–2000	[35]
Mauna Kea, Hawaii	68	6	8.8	2002–2004	[36]
Ottawa, Canada	20	1	5.0	2000	[33]
Prince Edward Island, Canada	96 185	5 14	5.2 7.6	2009 2001	[20] [34]
Merida, Mexico	227		2.5		[43]
St. Kitts, West Indies	99	15	15.2	2006–2007	[18]
St. Kitts, West Indies	72	10	13.9	2009	[18]
St. Kitts, West Indies	11	4	36.4	2010	[19]
St. Kitts, West Indies	105	18	17.1	2014–2015	This study
Grenada, West Indies	101	22	21.8	2004–2007	[41]
**Oceania**					
Sydney, Australia	68	15	22.1	2003–2004	[40]

## Data Availability

All data generated in this study are included in the manuscript.
